# Parvalbumin interneurons in Alzheimer's disease: Physiological insights and pathophysiological mechanisms

**DOI:** 10.1002/ccs3.70105

**Published:** 2026-08-10

**Authors:** Mengyan Wu, Xingdong Zeng, Yongle Cai, Haonan Chen, Qianying Li, Hao Yang

**Affiliations:** ^1^ Institute for Fetology The First Affiliated Hospital of Soochow University Suzhou Jiangsu China; ^2^ Shanghai First Maternity and Infant Hospital School of Medicine Tongji University Shanghai China; ^3^ Translation Medical Center Hong Hui Hospital Xi'an Jiaotong University Xi'an Shaanxi China

**Keywords:** Alzheimer's disease, gamma oscillations, neurological disorders, parvalbumin+ interneurons

## Abstract

Fast‐spiking parvalbumin‐positive (PV^+^) interneurons, a specialized class of inhibitory neurons, possess unique morphological and functional properties that govern spatiotemporal precision in local microcircuits, large‐scale network synchronization, and memory‐related computations. Since their initial identification in the late 19th century, technological innovations in cellular neuroscience have progressively elucidated the multifaceted roles of these neurons. In this review, we first delineate the embryonic origins and developmental trajectory of PV^+^ interneurons, emphasizing their unique properties for high‐frequency firing with remarkable temporal precision. These specialized features enable PV^+^ interneurons to orchestrate network oscillations and critically modulate memory encoding, consolidation, and retrieval, despite their substantial metabolic demands. We subsequently integrate multiple lines of evidence implicating region‐ and subtype‐specific PV^+^ interneuron impairment as a pivotal pathological hallmark in Alzheimer's disease (AD). Furthermore, we dissect molecular and cellular mechanisms driving PV^+^ interneuron dysfunction in AD, including numerical alterations, morphological remodeling, and electrophysiological disruptions. Critically, we propose that the pathological transformation of PV^+^ interneuron physiology emerges as a key driver in AD progression, bridging cellular dysfunction to system‐level cognitive failure.

## Graphical abstract

Physiological and Pathological Alterations in PV^+^ Neurons: Under physiological conditions, PV^+^ interneurons exhibit fast‐spiking properties and generate gamma oscillations at the cost of high energy consumption. In AD, PV^+^ neurons display abnormalities in ion channels, dendrites, neurotransmission, energy metabolism, and neural oscillations, ultimately leading to various neurological abnormalities.

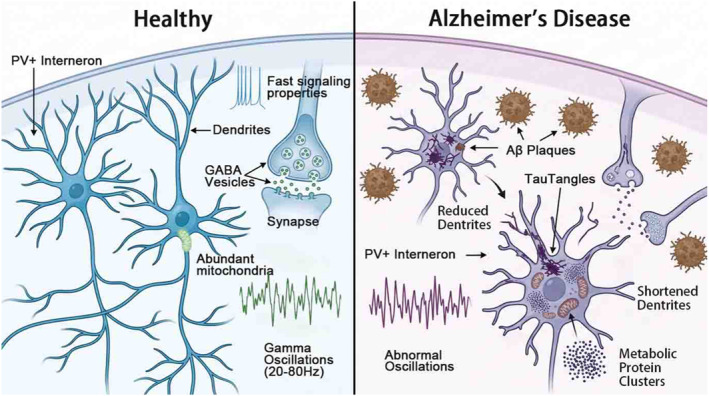

## 1 INTRODUCTION

The sophisticated computations underlying brain function arise from highly interconnected and dynamically regulated microcircuits consisting of two fundamental neuronal classes: (1) glutamatergic projection neurons that establish the excitatory backbone of neural networks, and (2) GABAergic interneurons that precisely modulate signal transmission and shape network dynamics. Although GABAergic interneurons constitute only 10%–15% of hippocampal neurons, they exhibit remarkable diversity and exert disproportionately powerful control over circuit function.^1^ Historically, GABAergic interneurons have been classified by neurochemical markers, such as parvalbumin (PV), calretinin (CR), somatostatin (SST), vasoactive intestinal polypeptide (VIP), neuropeptide Y (NPY), cholecystokinin (CCK), tyrosine hydroxylase (TH), ionotropic serotonin receptor 5HT3a (5HT3aR), and others. However, the most comprehensive catalog of neuronal diversity based on transcriptomics has revealed the existence of over 100 molecularly distinct subtypes of inhibitory neurons across mice, marmosets, and humans.^2^ Based on their distinct gene expression profiles from single‐cell sequencing, they are further classified into five groups: PV, SST, VIP, Lamp5, and Sncg.^2^, ^3^, ^4^, ^5^

Notably, PV^+^ interneurons constitute the largest subgroup, accounting for approximately 40% of all GABAergic neurons in many brain regions.^6^ Moreover, connections among cortical PV^+^ interneurons are more frequent than those among any other interneuron type, or between other interneurons and PV^+^ cells.^7^, ^8^, ^9^ They play a fundamental role in maintaining the cortical excitation–inhibition balance, as pyramidal neurons dynamically fine‐tune their inhibitory inputs specifically from this population.^10^ PV^+^ interneurons serve as master regulators of neural circuit operations: they fire high‐frequency action potentials (up to 300 Hz), release GABA with millisecond precision, and thereby synchronize gamma and theta oscillations while maintaining optimal E/I balance across neural networks.

Despite this remarkable functional homogeneity at the circuit level, PV^+^ interneurons are far from a uniform population. Within the hippocampal CA1 region, PV^+^ interneurons can be further subdivided into three major classes based on their morphological and synaptic targeting patterns: (1) PV^+^ basket cells (PVBCs) that form perisomatic synapses, (2) bistratified cells (BiCs) targeting dendritic compartments, and (3) axo‐axonic/chandelier cells (AACs) that specifically innervate axon initial segment (AIS).^11^ Additionally, a subset of oriens lacunosum‐moleculare interneurons (O‐LMs) also shows detectable PV expression though at substantially lower levels compared to the aforementioned PVBCs, BiCs, and AACs. Mounting evidence implicates PV^+^ interneuron dysfunction, whether through cellular loss, synaptic impairment, or metabolic compromise, as a key pathophysiological mechanism in several neuropsychiatric disorders, with particularly strong associations demonstrated in Alzheimer's disease (AD) pathogenesis.

In this comprehensive review, we systematically integrate contemporary understanding of fast‐spiking PV^+^ interneurons across multiple scales of biology from molecular signatures and cellular properties to network‐level integration, while emphasizing their region‐ and subtype‐specific heterogeneity. Our analysis focuses on three fundamental aspects: (1) the developmental origins and migratory pathways of PV^+^ interneurons, (2) their unique metabolic demands supporting high‐frequency firing, and (3) their diverse functional contributions to circuit operations. By synthesizing these perspectives, we construct a conceptual framework that bridges the physiological specialization of PV^+^ interneurons with their critical roles in information processing. This integrative approach not only elucidates the pathophysiological mechanisms underlying PV^+^ interneuron dysfunction in neurological disorders but also highlights emerging therapeutic opportunities, with particular emphasis on AD pathogenesis and potential intervention strategies.

## 2 ORIGIN AND DEVELOPMENT OF PV^+^ INTERNEURONS

**FIGURE 1:**
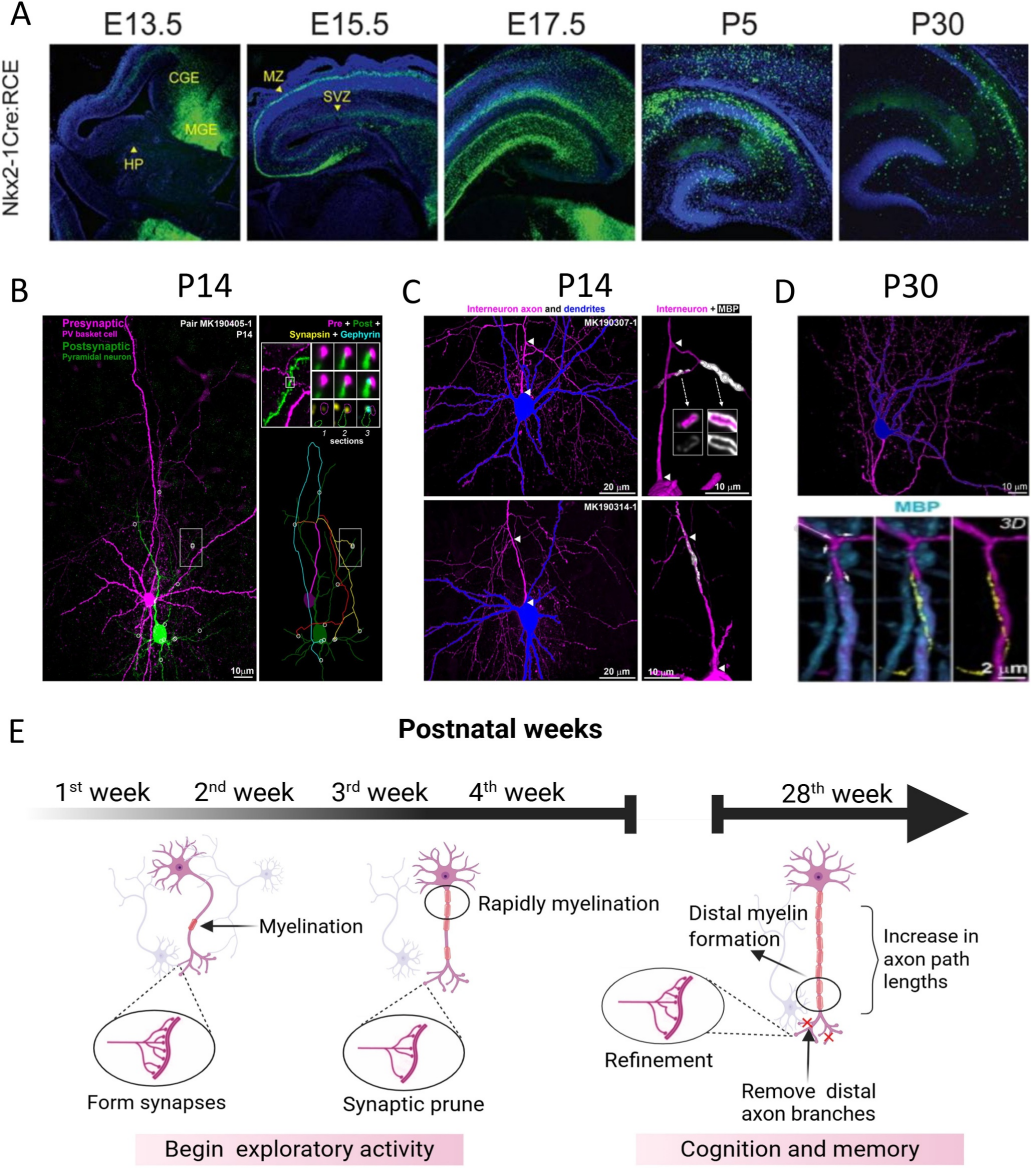
Development and maturation of PV^+^ interneuron: Neurogenesis, migration, and axonal and synaptic remodeling in neurons. (A) Representative images illustrating migration pattern of GFP‐labeled MGE‐derived interneurons in Nkx2‐1Cre:RCE line from E13.5 to P30. Adapted from.^12^ (B) Synaptic connectivity between a PV^+^ interneuron (magenta) and a pyramidal neuron (green) at P14. The inset shows serial sections of a synaptic contact (white circles) with presynaptic synapsin (yellow) and postsynaptic gephyrin (cyan). Adapted from.^16^ (C) The synaptic structure and myelination of PV^+^ neurons initiate around P14 (soma/dendrites in blue, axon in magenta). MAX projections through all sections from pairs MK190307‐1 (775 sections) and MK190314‐1 (375 sections). Arrow indicates the corresponding initial segment. Top right: partial myelination of tertiary branches with uneven MBP labeling; bottom right: incomplete myelination before and after the first branch point. Adapted from.^16^ (D) PV^+^ interneurons with the soma and dendrites in blue and axon in magenta from P30 neuronal pairs. Bottom panel shows axons bifurcation myelinated and unmyelinated branches at the second bifurcation point. Short arrow indicates the direction of the axon. Adapted from.^17^ (E) Schematic timeline of postnatal PV^+^ neurons. Weeks 1–2: PV^+^ neurons neuronal migration, initial synaptogenesis, and myelination onset. Weeks 3–4: rapid proximal axon myelination and synaptic pruning. Late postnatal period: PV^+^ neuronal axon elongation, distal myelination, branch refinement, and synaptic maturation, supporting the emergence of exploratory behaviors and cognitive flexibility.

The neocortical and hippocampal interneuron populations originate from progenitor zones in the medial and caudal ganglionic eminence (MGE and CGE). Extensive fate‐mapping studies have revealed that PV^+^ interneurons predominantly derive from the MGE,^12^, ^13^ with distinct subtypes showing specific spatial origins within this germinal region. In hippocampal CA1, PVBCs are the most abundant PV^+^ subtype, accounting for approximately 60% of all PV^+^ interneurons and originating from dorsal MGE progenitors. By contrast, AACs derive from the ventral‐most MGE domain,^14^ while BiCs, which constitute ∼6% of CA1 interneurons, similarly trace their lineage to MGE precursors.^12^, ^15^ The developmental journey of MGE‐derived PV^+^ interneurons involves precisely coordinated molecular and cellular events. Following their specification, these cells undergo extensive tangential migration to reach their final destinations in the hippocampus and neocortex. Genetic fate‐mapping using Nkx2‐1Cre:RCE transgenic mice has revealed that MGE‐derived interneurons follow distinct migratory pathways, traveling through both the marginal zone (MZ) and intermediate/subventricular zone (IZ/SVZ) streams (Figure 1A). The migratory and differentiation programs of these cells are orchestrated by a conserved transcriptional cascade. The LIM‐homeodomain transcription factor Lhx6 serves as a crucial downstream mediator of Nkx2.1 signaling in MGE‐derived interneuron development. Following their exit from the ventricular zone, MGE progenitors upregulate Lhx6 expression, which is subsequently maintained throughout maturation and into adulthood in the majority of MGE‐derived interneuron populations. Genetic ablation studies demonstrate that Lhx6 is indispensable for proper interneuron development, as its loss‐of‐function results in severe defects including impaired tangential migration and failure of PV fate specification.^18^ The transcription factor Sox6, acting downstream of Lhx6 in the genetic hierarchy, serves as a master regulator coordinating multiple developmental processes essential for PV^+^ interneuron ontogeny. Through its pleiotropic actions, Sox6 precisely controls: (1) directed migration along defined pathways, (2) cellular survival during critical developmental windows, (3) molecular specification of PV^+^ interneuron identity, and (4) functional maturation of intrinsic membrane properties and synaptic connectivity. These findings collectively highlight the existence of an evolutionarily conserved transcriptional cascade (Nkx2.1→Lhx6→Sox6) within MGE progenitor domains that orchestrates the sequential developmental program required for proper generation and circuit integration of PV^+^ interneurons, ultimately shaping the functional architecture of cortical inhibitory networks.

The developmental trajectory of PV^+^ interneurons extends from the embryonic stages through early postnatal life, culminating in a prolonged maturation process. Immunohistochemical studies reveal that parvalbumin‐expressing GABAergic neurons first emerge in specific cortical regions at postnatal days 8–9. In addition, they appear predominantly in Layers VI and IV, followed by Layers II and III.^19^ PV^+^ interneurons require an extended critical period spanning several months to achieve full electrophysiological and morphological maturity.^16^ During first postnatal week in murine neocortex, the majority of PV^+^ interneurons migrate to their final laminar destination and initiate synaptogenesis with local neuronal partners.^20^ This rapid integration phase culminates within 2 weeks, when PV^+^ interneurons either successfully incorporate into nascent cortical microcircuits or undergo programmed cell elimination (Figure 1B). Following the emergence of myelination (Figure 1C) and synaptic contacts in the first postnatal week, extensive refinement of inhibitory synapses and axonal paths in PV^+^ neurons has been observed in the following weeks.^16^ During postnatal weeks 3–4, PV^+^ interneurons undergo significant structural refinement characterized by rapid myelination of proximal axonal segments, and extensive synaptic pruning (Figure 1D). Quantitative analysis reveals a striking reduction in synaptic density, with the number of synapses per connection decreasing to approximately 33% of P14 values in the second half of the first postnatal month. Longitudinal observations from 2 weeks to 7 months of age demonstrate ongoing axonal remodeling, including selective elimination of distal axonal branches, and progressive standardization of axon path lengths accompanied by continued myelination.^16^ Collectively, PV^+^ interneurons exhibit a biphasic maturation pattern, transitioning from rapid early development to gradual refinement (Figure 1E). This extended developmental trajectory closely parallels the emergence of complex behavioral phenotypes.

PV^+^ interneurons are widely distributed across various brain regions, including the neocortex, hippocampus, thalamus, hypothalamus, cerebellum, and brainstem. These interneurons display regional heterogeneity in both morphology and function. The hippocampal CA1 region, the most extensively studied area, contains PV^+^ interneurons that are predominantly PVBCs and BiCs, with the remaining 15% being AACs.^11^, ^21^ The same is observed in the cerebral cortex.^22^, ^23^ In the neocortex, 15%–50% of AACs are PV‐positive, whereas in the hippocampus, PV‐negative AACs have not been reported. Beyond these subtype distribution differences, PV^+^ interneurons also exhibit region‐specific morphological heterogeneity in their axonal arbors and synaptic terminals. For example, striatal PV^+^ neurons exhibit compact, spherical axonal fields, whereas auditory cortex (AC) PV^+^ neurons form distinctive candle‐like synaptic terminals. Moreover, compared with AC‐PV neurons, tail‐of‐striatum (TS) PV^+^ neurons show extensively branched axonal processes.^24^ Functionally, PV^+^ interneurons also show region‐dependent innervation. In the neocortex, PVBCs preferentially innervate reciprocally connected pyramidal cells (PCs) that project to subcerebral targets (e.g., brainstem and spinal cord) rather than callosal ones, and evoke larger inhibitory postsynaptic currents (IPSCs). In the hippocampus, PVBCs elicit larger IPSCs in PCs near s.o. than those near s.r.^25^, ^26^, ^27^ PV^+^ interneurons also exhibit region‐specific integration patterns when innervating PCs. In the neocortex, they show sublinear integration, which is associated with a higher density of synapses on proximal dendrites and low NMDAR expression.^28^ In the cerebellum, PV^+^ interneurons on thin dendrites display passive, sublinear responses.^29^, ^30^ By contrast, hippocampal PV^+^ interneurons on thin dendrites exhibit both sublinear and supralinear synaptic integration.^31^, ^32^, ^33^ Furthermore, PV^+^ neurons exhibit region‐specific molecular expression: for example, CB1 receptors are expressed in striatal PV^+^ interneurons, whereas in the hippocampus, CB1 is restricted to CCK^+^ interneurons,^34^, ^35^ and hippocampal PV^+^ interneurons are enriched in μORs compared with their neocortical counterparts.^36^ Notably, neurochemical markers are traditionally considered mutually exclusive in the neocortex. However, recent evidence indicates that PV^+^ neurons can co‐express other neurochemical markers in a region‐ and species‐specific manner. In the human neocortex, 14.6% ± 5.6% of TH‐immunoreactive neurons are PV‐positive, and 28.0% ± 5.4% of CCK‐immunoreactive neurons also co‐express PV (confined to layer IV).^37^ In the mouse hippocampus, 40%–56% of PV^+^ interneurons in CA1 express CCK.^38^ In addition, a small proportion of PV‐expressing interneurons have been reported to transiently co‐express SST, as confirmed by dual immunostaining in Pvalb‐Cre and Sst‐Cre tdTomato reporter mice.^39^

## 3 FAST SIGNALING PROPERTIES OF PV^+^ INTERNEURONS

**FIGURE 2:**
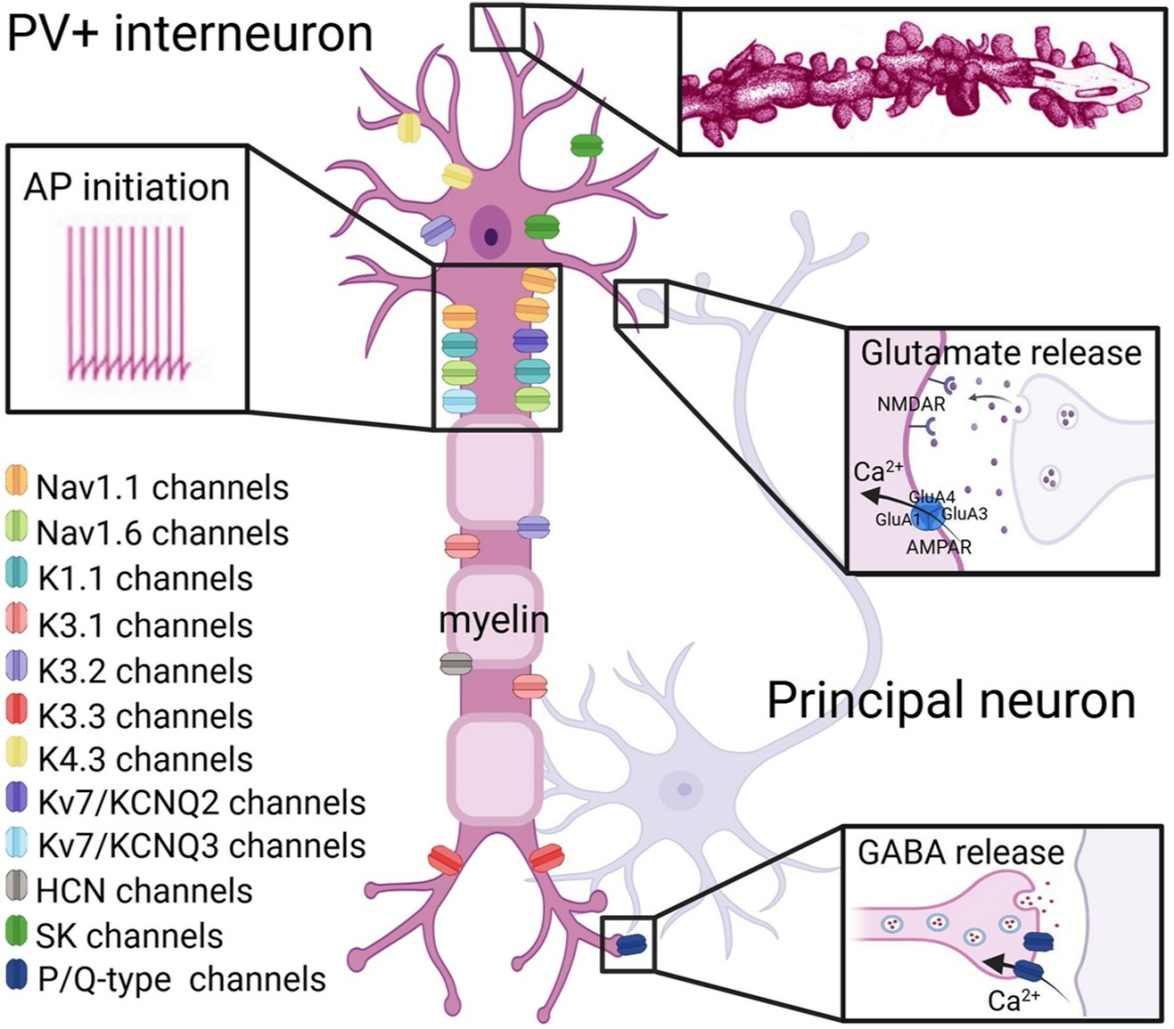
Ion channels and synaptic connections in PV^+^ neurons. The dendrites of PV^+^ interneurons receive dense synaptic inputs, while APs are initiated at the axon initial segment. Distinct ion channel subtypes, denoted by color‐coding, are strategically localized to regulate action potential generation and propagation. At synaptic terminals, PV^+^ neurons form specialized synaptic connections with principal neurons, employing dual neurotransmitter release: GABA and glutamate. At these synapses, P/Q‐type calcium channels governs Ca^2+^ influx for GABA release, while postsynaptic NMDARs and AMPARs bind the co‐released glutamate to depolarization and activate downstream signaling. Thereby, PV^+^ interneurons establish precisely and highly efficient inhibitory control over principal neuron activity.

Brain information processing relies on coordinated interactions between glutamatergic principal cells and GABAergic interneurons. PV^+^ fast spiking (FS) neurons are the most abundant population, characterized by their unique electrophysiological properties that govern temporal dynamics, neuronal ensemble formation, and network oscillations.^40^, ^41^ The FS phenotype of PV^+^ neurons arises from their electrophysiological properties, including exceptionally narrow APs and remarkably short refractory periods. When activated, these rapidly discharging PV^+^ neurons can sustain high‐frequency AP trains, enabling ultrafast GABAergic transmission through near‐synchronous neurotransmitter release from their presynaptic terminals with millisecond temporal precision. Subsequently, this fast inhibitory output is integrated by principal neurons, enabling the temporally precise control of excitatory output. These excitatory signals are subsequently detected by PV^+^ neurons through postsynaptic glutamate receptors, thereby completing a feedback loop between principal neurons and PV^+^ interneurons (Figure 2).

Synaptic inputs are fundamental prerequisites for AP generation in PV^+^ interneurons. These inputs rely on the intrinsic membrane properties of extensive dendritic arbor and soma, ultimately triggering AP initiation.^42^, ^43^, ^44^, ^45^ Notably, PV^+^ neurons possess exceptionally elaborate dendritic trees, with total lengths reaching >4300 μm.^46^ This morphological specialization supports extraordinary synaptic convergence: PV^+^ dendrites receive 6–8 times more synaptic contacts than other interneuronal subtypes,^46^ enabling rapid integration of excitatory postsynaptic potentials (EPSPs) (Figure 2). Voltage summation of these high‐density EPSPs precisely controls AP threshold dynamics. Crucially, AP initiation occurs at the AIS—a specialized microdomain proximal to the soma where integrated synaptic inputs reach the depolarization threshold for spike generation.^43^ This process is enabled by an approximately 60‐fold higher Na^+^ channel density in axonal membranes compared to somatic compartments and lower Na^+^ channel activation threshold than those interneurons.^43^ Molecularly, Na^+^ channel diversity enables compartment‐specific targeting in PV^+^ neurons, with Nav1.1 and Nav1.6 isoforms abundantly localized to axons (Table 1). These isoforms critically enable fast‐spiking properties through rapid activation kinetics and enhanced current density. The biophysical basis of ultrafast APs further involves coordinated voltage‐gated ion channel interactions: rapid Na^+^ channel inactivation coupled with delayed rectifier K^+^ channel activation generates metabolically efficient, submillisecond‐duration membrane potential.^43^, ^69^ This precisely timed interplay of conductances minimizes ionic flux per depolarization cycle while preserving spike amplitude fidelity. Furthermore, different kinds of Kv channels perform distinct functions and collaborate to promote membrane repolarization and support rhythm‐sustaining AP patterns. Kv1 channels contribute to delay‐type firing pattern and increase the threshold for APs, orchestrating with Kv3, Kv4, and Kv7 to modulate presynaptic AP duration and influence gamma oscillations (Table 1). Notably, the accelerated deactivation kinetics of Kv3‐type potassium channel subunits expressed in PV^+^ neurons support sustained high‐frequency firing by enabling rapid restoration of the resting membrane potential, thereby reducing inter‐spike interval variability.^70^ In addition, changes in AP waveforms at synaptic terminals play a critical role in regulating calcium influx and neurotransmitter release.^71^, ^72^ Coincidentally, Kv3 channels are particularly important in shaping presynaptic AP waveform. Recent studies indicate that the Kv3.3 subunits prolong presynaptic AP duration, enhance transmitter release, and reinforce short‐term depression upon repetitive stimulation.^73^ In contrast, the M‐current mediated by Kv7 channels is a slowly activating and weakly inactivating potassium current activated at subthreshold potentials, producing a steady voltage‐dependent outward current.^59^, ^60^, ^74^ Thus, the slow gating kinetics allows the M‐current to act as a brake for repetitive AP firing, thereby stabilizing membrane potential against depolarizing influences in the presence of depolarizing currents.

**TABLE 1:** Subcellular localization and physiological functions of diverse ion channels in PV^+^ interneurons.

Channels	Functional consequence	Location	Reference (s)
Nav1.1	Fast axonal APs; enhanced inhibitory synaptic currents	Proximal AIS regions	43, 44, 47
Nav1.6	Fast‐spiking Aps; higher firing capability; low‐threshold	Distal AIS regions	43
Kv1.1	Delay‐type firing pattern; increased the threshold current for Aps; decreased the firing frequency	AIS regions	43, 44, 48
Kv3.1	Repetitive, high‐frequency firing; fast repolarization	Nodes of Ranvier	49, 50
Kv3.2	Influences gamma oscillations; fast repolarization	Somata and axons	51, 52, 53
Kv3.3	Increased presynaptic AP duration and transmitter release; enhanced short‐term inhibition	Axonal terminals	54, 55, 56
Kv4.3	Mediated A‐type K^+^ currents and repetitive firing; modulated the AP shape	Somata and dendrites	55, 57, 58
Kv7/KCNQ2	Mediator of M‐type potassium current; modulated the AP shape	AIS regions	59, 60, 61
Kv7/KCNQ3	Mediator of M‐type potassium current; modulated the AP shape	Distal AIS regions	60, 61
Hyperpolarization‐activated cyclic nucleotide‐gated (HCN)	Enhanced AP initiation; facilitated the propagation of APs; counterbalanced the hyperpolarizing currents	Axons	62, 63
Small conductance calcium‐activated potassium (SK) channels	Lengthened individual Aps; enhanced after‐hyperpolarization potential	Somata and dendrites	64, 65, 66
P/Q‐Type calcium channels	Evoked neurotransmitter release	Presynapse	67, 68

Although these core biophysical properties are broadly shared, PV^+^ interneurons display region‐specific heterogeneity in their intrinsic electrophysiological properties. For example, tail‐of‐striatum PV^+^ neurons exhibit higher AP thresholds, lower firing rates, yet narrower AP waveforms with faster kinetics, deeper afterhyperpolarization, and a steeper F/I slope compared with AC PV^+^ neurons.^24^ Within the same brain region, dorsomedial striatum PV^+^ interneurons are more excitable than their dorsolateral counterparts, as evidenced by increased input resistance and a left‐shifted I‐V curve.^75^ These findings demonstrate that PV^+^ interneuron intrinsic properties are finely tuned by regional context and local circuit specialization, a dimension of functional diversity that must be considered when interpreting PV^+^ neuron contributions to circuit operations across different brain regions.

Irrespective of these subregional differences in somatic excitability, the ultimate functional output of PV^+^ interneurons is GABAergic synaptic transmission, which relies on the conversion of APs into presynaptic neurotransmitter release, a process governed by fast signaling properties. GABA release from the axons of PV^+^ interneurons is collectively determined by the properties of the fast signaling. The conversion from the electrical signals to GABA release depends on several presynaptic factors, including the duration of APs, the gating kinetics of the presynaptic Ca^2+^ channels, the coupling efficiency between Ca^2+^ channels and vesicular release sensors, and the Ca^2+^ binding/unbinding rates of the release sensor. Hyperpolarization‐activated cyclic nucleotide‐gated (HCN) channels, a type of cation channel permeable to Ca^2+^, generate the cationic Ih current in neurons and regulate neuronal network excitability (Table 1). In hippocampal circuits, HCN channels enhance the strength of evoked inhibitory synaptic transmission onto PCs. Blocking HCN channels reduces Ca^2+^ influx into the presynaptic boutons of PV^+^ interneurons and lowers the probability that a single electrical stimulus‐evoked AP evoking a calcium response.^62^ P/Q‐type Ca^2+^ channels also modulate GABA neurotransmitter release from PV^+^ neuron terminals. Inhibition of P/Q‐type Ca^2+^ channels similarly reduces the amplitude of the IPSCs in PV‐targeted principal neurons and suppresses the baseline GABA release probability, thereby diminishing presynaptic inhibitory output.^67^

The rapid output of PV^+^ interneurons is matched by specialized glutamatergic inputs that ensure ultrafast excitation. PV^+^ interneurons are exquisitely adapted for fast‐spiking activity, engaging in reciprocal circuits by receiving excitatory input from pyramidal neurons while providing rapid GABAergic synaptic feedback inhibition. This excitatory drive is shaped by a unique glutamate receptor profile, characterized by smaller N‐methyl‐D‐aspartate receptor (NMDAR) currents but larger calcium‐permeable α‐amino‐3‐hydroxy‐5‐methyl‐4‐isoxazolepropionic acid receptor (CP‐AMPAR) currents.^76^ Although both spine and dendritic synapses of PV^+^ neurons contain CP‐AMPARs and NMDARs, the latter are more abundant at spine synapses.^77^ AMPARs, which mediate most fast excitatory synaptic transmission, are tetrameric receptors composed of four subunits (GluA1‐GluA4).^78^ Calcium permeability in PV^+^ neurons arises primarily from the absence of the GluA2 subunit,^79^, ^80^, ^81^ as GluA2 incorporation effectively suppresses Ca^2+^ influx.^78^ Although GluA2 expression in PV^+^ neurons remains somewhat debated, prevailing evidence supports a predominant GluA2‐deficient composition.^78^, ^82^, ^83^ The accelerated AMPAR kinetics observed in these interneurons, compared to principal neurons, are likely attributable to their higher expression of the GluA4 subunit.^84^, ^85^, ^86^ Notably, APs initiation in PV^+^ neurons exhibits a significantly greater reliance on AMPAR‐mediated excitation than on NMDAR‐mediated excitation.^87^ This is partly because, near resting or depolarized subthreshold potentials, NMDAR channels are largely blocked by Mg^2+^, whereas AMPAR channels lack this voltage‐dependent block.^88^ Consistent with their fast signaling role, PV^+^ neurons exhibit a shorter‐duration excitatory postsynaptic currents, a trait attributed to GluA2‐lacking AMPARs.^87^, ^89^ In contrast, NMDARs display high Ca^2+^ permeability, crucial for triggering synaptic plasticity through Ca^2+^/calmodulin‐dependent kinase IIα (CaMKIIα) activation.^88^ Their voltage‐dependent Mg^2+^ block prevents NMDARs from independently initiating excitation without depolarization via AMPARs or voltage‐gated channels. Upon activation, however, NMDAR currents last substantially longer (∼100–400 ms) than AMPAR‐mediated responses (∼2–10 ms).^88^ Analogous to AMPAR kinetics, the shortest NMDAR‐EPSCs are governed by GluN2A‐containing subunit.^88^, ^90^ Overall, the elevated AMPAR/NMDAR ratio and specific subunit composition of PV^+^ neurons optimize their glutamatergic synaptic inputs for ultrafast activation, underpinning their capacity for high‐frequency firing and precise inhibitory control.

## 4 PV^+^ NEURONS INVOLVED IN GAMMA OSCILLATIONS

**FIGURE 3:**
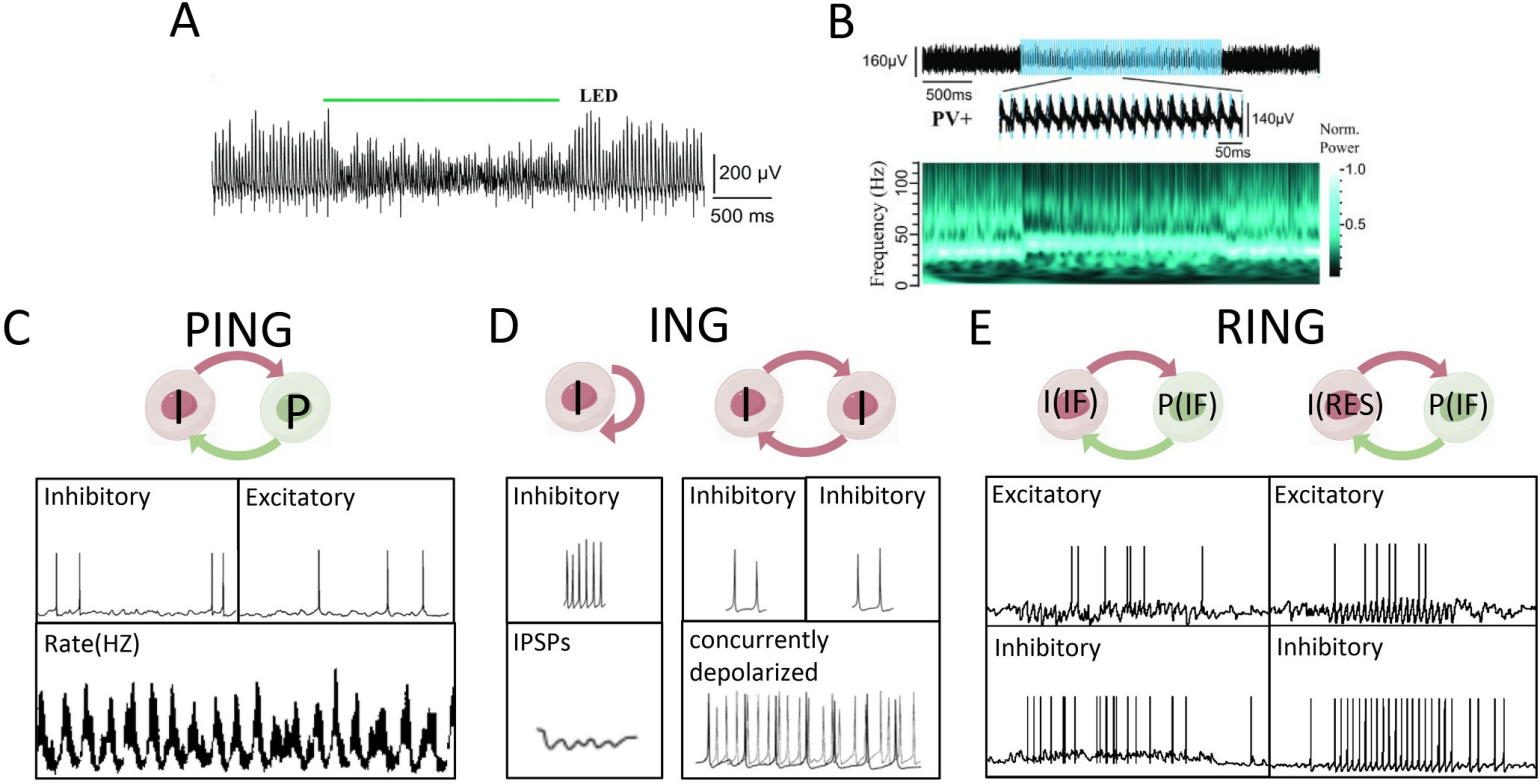
PV^+^ neurons in gamma oscillation mechanisms. (A) Suppression of cholinergically induced oscillations during optogenetic inhibition of PV^+^ interneurons. Adapted from.^95^ (B) Entrainment of carbachol‐induced oscillations to 40 Hz optical stimulation in PV‐Cre mice expressing mCherry‐ChR2. Adapted from.^95^ (C) Schematic of the pyramidal‐interneuron gamma mechanism. Top: pyramidal cells (PCs) provide rapid excitation to interneurons, which reciprocally inhibit PCs. Bottom: resting coherent gamma‐frequency oscillations. Adapted from.^96^ (D) Schematic diagram of the interneuron gamma mechanism. Top left: depolarization of a single inhibitory interneuron. Bottom left: resulting inhibitory postsynaptic potential in a synaptically connected interneuron. Top right: simultaneous depolarization of two inhibitory interneurons. Bottom right: population‐level activity showing superimposed membrane potentials (black) and single‐cell traces (gray) during synchronous firing. Adapted from.^97^ (E) Schematic of the resonance induced gamma mechanism. Left: membrane potential traces of two neurons in an IF‐IF network. Right: membrane potential traces for two neurons from an IF‐RES network, showing enhanced rhythmicity. Adapted from.^98^

PV^+^ neurons regulate network oscillations and contribute to memory encoding, consolidation, and retrieval, which is supported by their high‐frequency firing capability and synaptic reliability.^91^ These cells are specialized for rapid network synchronization, resonating at gamma frequencies and delivering powerful perisomatic inhibition that precisely controls spike timing in principal neurons. Gamma oscillations (30–80 Hz), a biomarker of cognitive performance, arise from rhythmic and synchronous fluctuations of the membrane potential of neuronal populations, providing a substrate for cell‐to‐network plasticity. When a sufficient number of neurons were fire within a narrow time window, they generate a substantial inhibitory conductance across the network. This rapid inhibition enables PV^+^ neurons to integrate and synchronize excitatory inputs from pyramidal neurons.^92^, ^93^, ^94^ Consistent with this role, disrupting PV^+^ neurons activity, for example, via optogenetic inhibition, typically reduces slow gamma power, whereas optogenetic activation of PV^+^ neurons is sufficient to initiate and sustain gamma oscillation^95^ (Figure 3A,B).

With regard to PV^+^ neuron‐mediated gamma oscillations, the mechanisms are primarily involved in mutually connected inhibitory interneurons, the time constant conferred by GABA receptor‐mediated inhibition, and sufficient excitatory drive to triggering interneuron spiking.^92^ Notably, gamma oscillations vary across brain regions in terms of frequency, power, and network coherence. For instance, hippocampal gamma (25–100 Hz) and neocortical gamma (40–160 Hz) exhibit distinct spectral properties and laminar profiles, suggesting that regional heterogeneity in PV^+^ interneuron connectivity and synaptic dynamics may shape oscillation properties.^99^, ^100^, ^101^, ^102^ Current research has classified gamma oscillation generation into three principal mechanisms: pyramidal‐interneuron gamma (PING), interneuron gamma (ING), and resonance induced gamma (Figure 3).^98^ Although gamma oscillations are widespread across brain regions, most mechanistic insights derive from hippocampal studies. In the PING model, oscillations originate in CA3 and propagate to CA1. Hippocampal fast network oscillations are associated with alternating current sink‐source pairs in CA3 stratum pyramidale and stratum radiatum, resembling those observed during in vivo gamma activity.^81^, ^103^ In this mechanism, pyramidal neurons fire at low frequencies (<5 Hz), phase‐locked to stratum pyramidal current sink, while receiving rhythmic perisomatic GABAergic inputs from interneurons to sustain oscillations (Figure 3C).^104^, ^105^ AMPAR‐mediated fast recurrent excitation from PCs then synchronizes the perisomatic‐targeting interneurons, thereby promoting fast network oscillations.^81^ Therefore, PING relies on a synaptic feedback loop between PCs and PV^+^ interneurons. In contrast, the ING model emphasizes mutual inhibition among interconnected inhibitory interneurons, which ultimately generates synchronized gamma frequency oscillations.^97^ Two interconnected inhibitory interneurons mediate synaptic activity and generate membrane potential responses via GABA receptors. When one interneuron depolarizes and fires a high frequency train (>100 Hz), it induces a temporally summed inhibitory postsynaptic potential in its connected partner (Figure 3D). Simultaneous depolarization of both interneurons leads to reciprocal inhibition and synchronous gamma‐frequency firing (Figure 3D).^97^, ^106^ Another important mechanism related to oscillations is the intrinsic frequency preference of neurons, also called membrane resonance.^107^ Despite the well‐known capacity of inhibitory interneurons, especially PV^+^ FS cells, to exhibit frequency preference in the gamma band (20–80 Hz), membrane resonance remains relatively understudied in the context of fast oscillations.^108^, ^109^ Membrane resonance depends on multiple voltage‐gated ion channels and is subject to neuromodulatory influence. Based on interneuron subtypes, resonance networks were categorized into two configurations: IF–IF (excitatory IF–inhibitory IF) and IF–RES (excitatory IF–inhibitory RES). The latter exhibits more regular and powerful oscillatory activity (Figure 3 E).^98^ By enhancing the entrainment of neuronal firing to specific frequencies, membrane resonance significantly increases the stability, robustness, and power of gamma oscillations.

PV^+^ interneurons are intrinsically equipped to participate in gamma oscillations due to a unique combination of numerical prevalence, anatomical specialization, and biophysical properties. First, as the most abundant GABAergic subtype comprising approximately 40% of cortical inhibitory neurons, they are positioned to exert broad influence on the neural network.^6^ Second, their distinctive axonal “baskets” innervating the perisomatic region of pyramidal neurons, enable powerful and synchronous control of output firing. This is supported by dendritic integration properties that allow PV^+^ cells to convert inputs from diverse principal neurons into coherent rhythmic output.^110^ Third, gamma oscillations are associated with alternating current sources and sinks in the perisomatic region, while this characteristic is consistent with the morphological structure of the PV^+^ neurons that innervate this subcellular domain.^1^, ^81^, ^103^ Upon activation, these PV^+^ interneurons generate rapid current sinks via photocurrents and APs, followed by GABAergic source currents, directly contributing to extracellular field oscillations.^98^, ^111^ Finally, fast‐spiking phenotype AP, with high‐frequency firing capability and intrinsic resonance, is ideally suited for pacemaking in the gamma band. These PV^+^ interneurons respond robustly to sensory stimuli, and their gamma‐range synchronization is thought to enhance perceptual precision by temporally aligning the discharge of principal cells following sensory input.^112^, ^113^, ^114^ Notably, these contributions are subtype‐dependent: PV^+^ basket cells, through their dense perisomatic innervation, are considered primary drivers of gamma oscillations, whereas axo‐axonic cells may contribute to phase resetting and cycle‐by‐cycle refinement of gamma activity.

## 5 HIGH ENERGY UTILIZATION IN PV^+^ NEURONS

**FIGURE 4:**
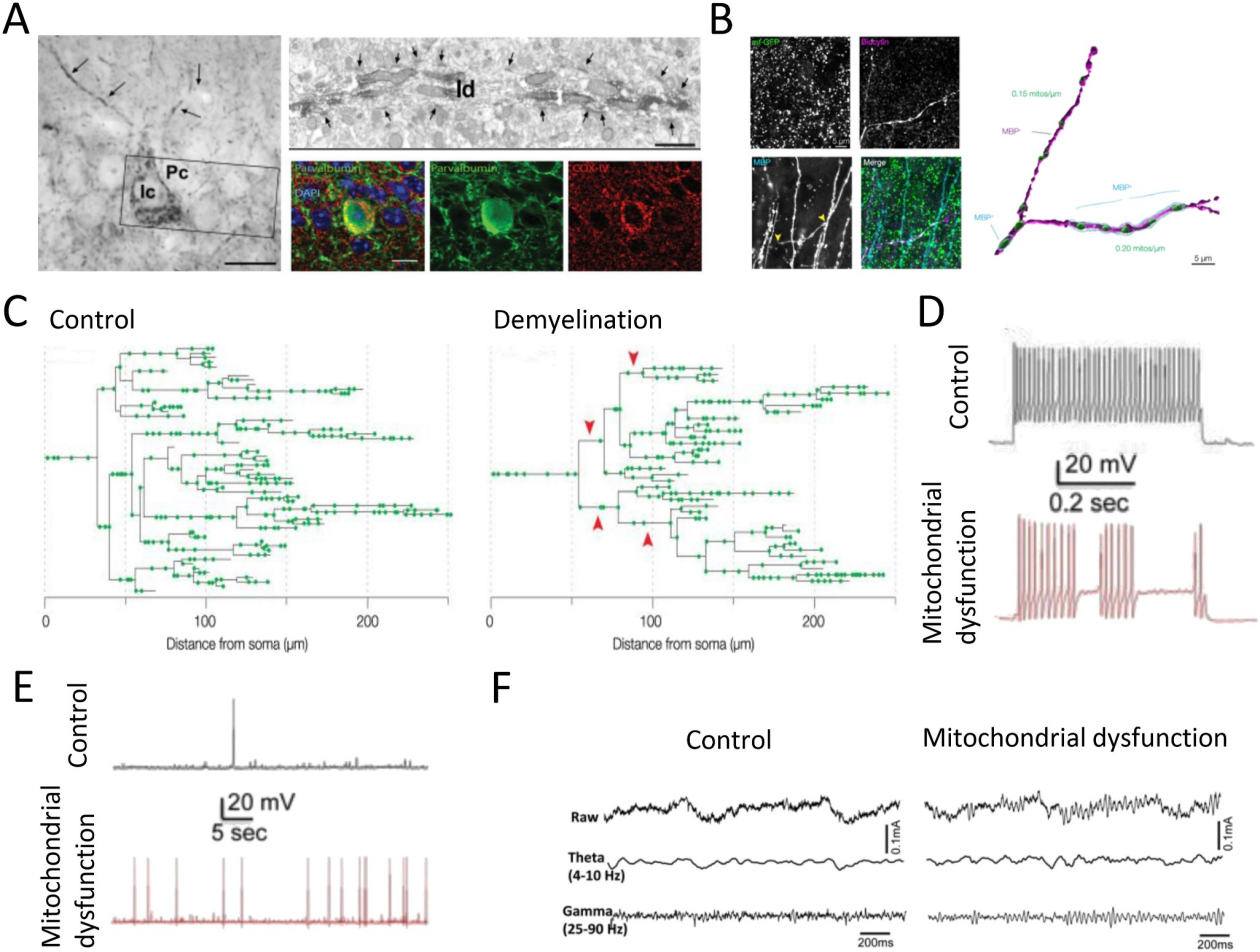
Mitochondrial distribution supports energy metabolism in PV^+^ neurons. (A) Enhanced mitochondrial density in GABAergic neurons. Immunoperoxidase reaction with 3,3′‐diaminobenzidine‐4HCl shows stronger cytochrome c (CC) labeling in inhibitory neurons (Ic) compared to pyramidal cells (Pc). Top right: PV^+^ dendrites in stratum radiatum are densely packed with mitochondria (Id, arrows indicate synaptic inputs). Bottom right: elevated CC oxidase (COX‐IV) levels in PV^+^ neurons. Adapted from.^120^, ^121^ (B) Axonal mitochondrial distribution at branch points. Confocal imaging and 3D reconstruction show a PV^+^ axon bifurcating into one myelinated (MBP^+^) and one unmyelinated (MBP^−^) branch. Adapted from.^122^ (C) Altered mitochondrial patterning following demyelination. Axonograms illustrate reduced mitochondrial density at higher‐order branches (red arrowheads) in demyelinated PV^+^ interneurons versus controls. Adapted from.^122^ (D, E) Representative electrophysiological impact of mitochondrial dysfunction. (D) Increased firing adaptation and (E) elevated spontaneous firing in PV^+^ interneurons from mice with mitochondrial impairment (red) compared to controls (black). Adapted from.^123^ (F) Altered network oscillations in mitochondrial‐deficient mice. Raw local field potential traces and filtered theta/gamma oscillations in medial prefrontal cortex at P60. Adapted from.^123^

The fast‐signaling properties of PV^+^ neurons and their engagement in gamma oscillations are functionally coupled to an exceptionally high energy demand. First, fast‐spiking capability enables high‐frequency AP firing. AP generation is energetically costly, as the flux of Na^+^ and K^+^ ions during each spike dissipates transmembrane gradients, necessitating active restoration by Na^+^/K^+^ ATPase.^115^ Further, PV^+^ neurons produce spikes at a much higher frequency than the principal neurons.^116^ The high energy demand of their brief spikes is due to the short APs associated with a large K^+^ conductance during the repolarization phase, resulting in overlapping Na^+^ and K^+^ fluxes during this period.^69^ These counterfluxes do not lead to changes in membrane potential but need to be reversed by Na^+^/K^+^ ATPase activity.^117^ In addition, the high‐frequency rhythmic activity of PV^+^ interneurons underlies gamma oscillations, during which peak oxygen consumption approaches levels observed during seizures, pushing mitochondrial oxidative capacity toward its functional limit.^118^ This elevated metabolic demand is accompanied by rapid shifts in cellular redox state and adaptations in glycolysis and oxidative phosphorylation pathways.^119^ Therefore, this high energy requirement of PV^+^ interneurons puts them into a pivotal position for the sustaining or compromising of cognitive functions under metabolic stress. To support this demanding functional profile, PV^+^ interneurons exhibit specialized metabolic adaptations. Approximately 75% of PV^+^ axonal boutons are mitochondria‐enriched, compared to fewer than half in pyramidal axon terminals.^120^ PV^+^ neurons also express higher density of cytochrome C (CC) and CC oxidase (CO), key components of the oxidative phosphorylation chain, than pyramidal neurons (Figure 4A). In this pathway, CC shuttles electrons to CO (complex IV), ultimately driving ATP synthase activity.^121^ These features reflect a cellular compromise between maximizing information processing speed and minimizing energy expenditure, positioning PV^+^ interneurons at the metabolic core of cortical computation.

Information processing in the nervous system requires a highly organized neuronal architecture, with synapses distributed at varying distances from the soma. To meet localized energy demands, mitochondria are strategically positioned to support ATP production and calcium buffering at active sites. The spatiotemporal distribution of mitochondria is thus critical for neuronal transmission and communication. Neuronal organelles form a highly dynamic and adaptable network, with considerable heterogeneity in sizes and morphology,^124^ and approximately one‐third of axonal mitochondria are motile, transporting at velocities ranging from 0.2 to 2 μm/s.^125^ PV^+^ interneurons rapidly activated inhibitory cells that regulate local circuit activity and oscillation, possess extensive axonal arbors.^41^ This extensive arborization makes the regulated transport and positioning of mitochondria particularly important in these cells. Notably, PV^+^ axons are frequently myelinated, and myelination influences mitochondrial function and morphology. Single‐cell reconstructions have shown that mitochondria preferentially accumulate in myelinated segments of PV^+^ basket cells, while demyelinated axonal regions exhibit approximately 20% lower mitochondrial density (Figure 4B,C).^122^ Proper mitochondrial trafficking and spatiotemporal localization are closely linked to synaptic integration and response to excitatory inputs. Disruption of mitochondrial distribution leads to enhanced network excitation, characterized by increased excitatory drive onto PV^+^ interneurons.^120^

Due to their high energy demand, PV^+^ neurons are particularly susceptible to mitochondrial dysfunction. As these PV^+^ neurons rely heavily on oxidative metabolism, impairments in mitochondrial function disrupt their intrinsic excitability, synaptic connectivity as well as network synchronization, ultimately compromising gamma oscillations and higher‐order information processing, and contributing to neuropsychiatric disorders.^118^, ^123^, ^126^ In mice with mitochondrial defects, PV^+^ neuron failed to sustain AP firing, as reflected by higher variability and a three‐fold increase in spike frequency adaptation (Figure 4D). However, PV^+^ neurons are more excitable with an increase in spontaneous firing and relative power of both gamma and theta oscillations (Figure 4E,F).^123^ These electrophysiological disruptions are accompanied by cognitive and social deficits, including impaired cognitive flexibility, memory retention, social interaction, and novelty discrimination, which are likely mediated by oxidative stress‐induced mitochondrial dysfunction.^126^, ^127^

## 6 PV^+^ INTERNEURON DEFICITS IN NEUROLOGICAL AND ALZHEIMER'S DISEASES

Given the morphological and fast‐signaling properties of PV^+^ neurons, they play a critical role in advanced computations in microcircuits and neuronal networks. Dysfunction of PV^+^ neuron has been extensively linked to multiple brain diseases, including AD, schizophrenia, epilepsy, and autism, all sharing phenotypes of network instability, reduced synaptic plasticity, and memory impairments. AD, the most prevalent neurodegenerative dementia, affects approximately 55.2 million people worldwide.^128^ Thus, AD is arguably one of the most prevalent and challenging neurological diseases. The clinical manifestations and pathogenesis are highly complex: early stages may be associated with epilepsy, depression, and olfactory dysfunction, while progressive cognitive and memory decline define later disease outcomes.^129^, ^130^, ^131^, ^132^, ^133^ Although numerous genetic risk factors and early biomarkers for AD have been identified, the core pathogenic mechanisms driving underlying cognitive impairment in AD remain highly controversial. Emerging evidence points to neuronal hyperexcitability as an early feature in both familial and sporadic AD models, during the prodromal disease stages.^134^, ^135^, ^136^ Similarly, aberrant brain activity is observed in humans with mild cognitive impairment and early‐stage familial AD.^137^, ^138^ Notably, increases in circuit activity also may directly accelerate pathology by promoting toxic protein deposition. As key regulator of excitatory–inhibitory balance, PV^+^ interneurons strategically positioned to influence AD progression. Selective impairments in PV^+^ interneuron physiology have been documented across multiple AD models and appear to causally contribute to early cognitive deficits.^139^, ^140^ Across clinical variants, a recurring pathomechanism involving PV^+^ neurons is PV^+^ neuron‐mediated circuit dysfunction, underscoring their central role in disease pathogenesis.

The regulatory cellular morphology and function of PV^+^ neurons in neural microcircuits depends critically on their structural and functional integrity. In AD, the neurons undergo progressive morphological and molecular alterations. First, ion channel abnormalities emerge as an early pathological event. In transgenic hAPPJ20 mice, reduced expression of the sodium channel subunit Nav1.1 on PV^+^ interneurons impairs inhibitory synaptic transmission.^140^ Nav1.6 levels also demonstrate a declining trend in AD models.^141^ Concurrently, Kv3 potassium channels exhibit biophysical abnormalities that compromises AP generation in PV^+^ interneurons and contributes to cortical hyperexcitability in AD mice.^142^ Second, structural reorganization of PV^+^ neurons evolves throughout AD progression. In 9–12‐month rat AD models, PV^+^ interneurons display increased dendritic length and elevated dendritic complexity.^143^ Although PV^+^ neurons density remains unchanged, dystrophic terminals proximal to amyloid plaques become more abundant, and the synaptic territory around the AIS significantly expands.^144^ Enhanced PV^+^ interneuron synaptic innervation is already detectable in the hippocampus of 3‐month‐old APP/PS1 mice.^145^ Notably, by 12 months, plaque accumulation within PV^+^ interneurons coincides with declining dendritic complexity,^146^, ^147^ progressing to significant dendritic arbor simplification and shortening by 15 months.^147^ Third, metabolic adaptations emerge as a distinctive response.^148^ Contrary to the overall synaptic impact during early and mid‐stages of AD, PV^+^ neurons showed a marked upregulation of mitochondrial and metabolic proteins in the late stages of the disease. This upregulation, affecting mitochondrial functional proteins and subunits of respiratory complexes I, III, IV, and V, may represent a compensatory mechanism to sustain energy demands, potentially triggered by Aβ interactions or early mTOR signaling dysregulation. Finally, neuronal loss marks advanced pathology, with significant reductions in PV^+^ interneuron density observed in hippocampal subfields CA1, CA3, and the dentate gyrus of AD models.^149^, ^150^, ^151^, ^152^

In AD, PV^+^ interneurons undergo progressive functional decline that parallels their structural pathology. Studies in AD mouse model reveal an age‐dependent trajectory of PV^+^ interneuron excitability: initial hyperexcitability at early stages (around 4 months) gives way to progressive synaptic dysfunction, characterized by reduced excitatory input and impaired inhibitory output, culminating in network hypoactivation by later disease stages (approximately 6 months). For example, PV^+^ neurons in the entorhinal cortex and subiculum exhibit early hyperactivity and contribute to Aβ pathology.^153^, ^154^, ^155^ However, this early dysfunction eventually gives rise to more widespread synaptic impairments. Short‐term plasticity in PV^+^ interneurons play a key role in dynamically regulating synaptic strength and timing, which is critical for maintaining precise E/I balance and for supporting neuronal oscillations. A consistent finding across AD models is E/I imbalance, characterized by an elevated sEPSC/spontaneous inhibitory postsynaptic current (sIPSC) charge transfer ratio accompanied by significant reductions in sIPSC frequency and charge per event (area).^139^ Hippocampal multi‐electrode recordings further demonstrate that Aβ oligomers reduce the amplitude of sIPSCs, weakening PV^+^ interneuron‐mediated inhibition of CA1 PCs and suppressing the theta and gamma oscillations.^139^ At the synaptic level, Aβ oligomers induce presynaptic dysfunction at PV‐to‐PC synapses by increasing initial GABA release, leading to frequency‐ and cell type‐specific suppression of PV‐evoked IPSCs.^156^ These impairments in inhibitory input likely desynchronizes CA1 PC spiking, thereby contributing to the diminished oscillatory capacity observed in Aβ oligomer‐injected mice.^156^

### 6.1 Region‐selective vulnerability of PV^+^ interneurons in Alzheimer’s disease

PV^+^ interneuron vulnerability is not uniform across the brain, but rather exhibits both region‐selective and subtype‐specific patterns. Regional susceptibility is already evident in early‐stage pathology. In the entorhinal cortex, one of the first regions to exhibit neurodegeneration and hyperexcitability in AD, amyloid overproduction drives PV^+^ interneuron hypoexcitability as early as 2 months of age, whereas PV^+^ cells in the relatively spared primary visual cortex remain largely unaffected.^157^ This vulnerability is not merely a consequence of local pathology but appears inherent to PV^+^ interneurons themselves, as entorhinal hyperexcitability results from selective vulnerability of PV^+^ interneurons with respect to surrounding excitatory neurons.^153^ Notably, this regional susceptibility extends to the retrosplenial cortex (RSC), another early‐affected region where PV^+^ interneurons are particularly vulnerable.^158^ Transcriptomic analyses reveal that RSC PV^+^ interneurons are not homogeneous but segregate into distinct subpopulations with differential vulnerability to AD‐related stress, showing coordinated downregulation of genes essential for metabolic function (Cox6a2), calcium buffering (Pvalb), GABAergic signaling (Gad1), and dendritic integrity (Dner).^158^ This transcriptional heterogeneity is accompanied by sex‐dependent effects, with female mice showing greater PV^+^ interneuron vulnerability in the RSC, a pattern mirrored by sex‐dependent RSC functional connectivity changes in humans with mild cognitive impairment and AD.^159^ Beyond the RSC and entorhinal cortex, the subiculum, the earliest hippocampal anatomical marker of AD, exhibits a distinct pattern: PV^+^ interneurons here show pronounced hyperactivity during initial disease stages and actively drive early Aβ pathology and cognitive deficits.^155^ Targeted inhibition of this hyperactivity reduces Aβ accumulation and restores cognitive function. Similarly, the prefrontal cortex (PFC) is selectively vulnerable in early AD, where PV^+^ interneuron deficits occurr much earlier than hippocampal impairments.^160^ Importantly, regional vulnerability is further refined by subtype specificity: even within the same region, distinct morphological subtypes show differential susceptibility. In the hippocampus, PV^+^ basket cells, but not bistratified or axo‐axonic cells, show selectively reduced activity during sharp‐wave ripples, a network event critical for memory consolidation.^153^ Furthermore, region‐specific vulnerability is also reflected at the molecular level: entorhinal cortex PV^+^ interneurons are enriched in proteins associated with cognitive resilience that are depleted in human AD, whereas somatosensory cortex PV^+^ interneurons show higher levels of tau interactors.^153^ Collectively, these findings establish that PV^+^ interneuron vulnerability in AD is governed by both anatomical location and cell‐type identity, highlighting the need for region‐ and subtype‐targeted investigations and early interventions that may protect vulnerable regions from downstream cognitive decline.

### 6.2 Pathophysiological implications of PV^+^ interneuron deficits in Alzheimer's disease

#### 6.2.1 Memory deficits

PV^+^ interneurons play critical roles in multiple memory modalities, including spatial, fear, and social memory.^139^ Memory deficits in AD are rooted in an E/I imbalance, defined as a disrupted equilibrium between glutamatergic and GABAergic synaptic inputs.^161^, ^162^ This imbalance constitutes a violation of a core prerequisite for normal brain function. Excessive glutamatergic transmission is considered a key contributor to this E/I imbalance in AD. Given their role in precisely and rapidly regulating pyramidal neurons in the hippocampus and cortex, PV^+^ interneurons have emerged as a focus of AD research.

Recent evidence suggests that memory deficits in AD models correlate with PV^+^ interneuron loss and dysfunction.^139^, ^160^ These models show reduced numbers of PV^+^ interneurons, decreased immunofluorescence intensity for PV, and lowered activity in the prefrontal cortex (PFC), dorsal CA1, and CA2/CA3 hippocampal subfields.^139^, ^150^, ^160^ Consistent with this, immunohistochemical analysis of human AD brain reveals approximately 60% loss of PV^+^ interneurons in the DG/CA4 and CA1/CA2 regions.^163^ Notably, optogenetic or chemogenetic activation in the PFC can rescue short‐term spatial memory and ameliorate depression‐like behaviors in young adult AD mice,^160^ highlighting the therapeutic potential of targeting PV^+^ circuits to restore E/I balance and cognitive function.

Memory impairment in AD is also attributed to widespread synaptic weakening and loss.^164^ These synapses form the crucial connections that specifically link PV^+^ interneurons to pyramidal neurons to control fast‐spiking and modulate network excitability in hippocampus and cortex. In AD, the impairment of synapses is partly attributable to reduced Nav1.1 expression in PV^+^ neurons. Aβ deposition downregulates this voltage‐sensitive sodium channel Nav1.1 in PV^+^ inhibitory interneurons, leading to hyperexcitability of pyramidal neurons.^141^ Furthermore, memory deficits associated with synapses involve the inhibitory terminals of PV^+^ neurons, where GABA is released onto cortical pyramidal neurons. The loss of PV^+^ neurons consequently reduces GABAergic transmission, even though GABA synthesis and transport remain intact in AD mice.^160^ Synaptic dysfunction in AD is further exacerbated by the ablation of NMDARs and decreased expression of the AMPAR subunit GluA4 on PV^+^ interneurons.^164^, ^165^ As GluA4 is preferentially localized at excitatory synapses on PV^+^ interneurons and enable rapid channel kinetics, its downregulation undermines the fast‐spiking capacity of these interneurons and compromises their critical contribution to hippocampal rhythmicity and memory process.

#### 6.2.2 Anxiety and depression

Anxiety and depression are common prodromal manifestations of AD. Notably, analogous anxiety‐like comorbid phenotypes have been observed in AD rodent models, highlighting their relevance as early indicators of disease progression.^151^ Major depressive disorder is recognized as an independent risk factor for subsequent AD development,^129^, ^131^, ^160^ and greater anxiety severity correlates with accelerated cognitive decline in patients.^166^ In AD mouse models, anxiety‐like behaviors observed are linked to hippocampal E/I imbalance, likely resulting from reduced PV^+^ interneuron density in this region. The loss of PV^+^ interneurons diminishes inhibitory control over principal neurons, leading to network hyperexcitability. Importantly, selective inhibition of hippocampal PCs normalizes both anxiety‐like behaviors and E/I balance in AD mice.^151^ Conversely, targeted activation of hippocampal PV^+^ interneurons similarly ameliorates anxiety‐like behaviors and restores synaptic E/I balance in the CA1 region.^151^, ^160^ At the molecular level, GABA receptors on PV^+^ interneurons in hippocampal CA1 and DG undergo age‐dependent alterations, characterized by a progressive decline in the δ subunit expression, a deficit consistently observed in postmortem human AD and transgenic AD mouse models. Positive allosteric modulation of δ‐containing GABA receptors ameliorates anxiety‐like behaviors in AD mice,^166^ suggesting a potential therapeutic pathway for early AD‐related neuropsychiatric symptoms.

#### 6.2.3 Epilepsy

Epileptic activity is increasingly recognized as a comorbidity of AD, usually emerging in early stages and potentially accelerating disease progression. Seizures in AD patients are associated with more rapid cognitive decline, highlighting the clinical importance of early detection and intervention. However, clinical identification remains challenging since seizures in AD frequently present as nonconvulsive episodes that can be masked by other disease manifestations. PV^+^ interneurons are primarily responsible for maintaining network stability through fast perisomatic inhibition of principal excitatory neurons. Their impairments leads to pathological neuronal synchronization, promoting the initiation and spread of seizure activity. Recent proteomic analyses of PV^+^ interneuron‐enriched have identified significantly elevated levels of specific proteins, particularly syntaxin‐1B (Stx1b) and GABA transporter 1 that are implicated in both epileptogenesis and AD pathology.^148^ Accumulation of tau protein within PV^+^ interneurons represents another mechanism contributing to AD‐related epileptogenicity. In 15‐month‐old AD model rats, PV^+^ interneurons show cytoplasmic tau inclusions accompanied by reduced dendritic length and diminished PV immunoreactivity.^147^ Strikingly, in contrast to the 15‐month phenotype, 9‐month‐old animals exhibit different morphological changes, suggesting a biphasic phase that PV^+^ interneurons initially undergo compensatory adaptations before progressing to overt dysfunction. Additional mechanism includes a reduction in NaV1.1 channel expression in PV^+^ interneurons, which impairs their firing capacity and disrupts hippocampal gamma oscillations.^152^, ^167^ Correspondingly, decreased synaptic charge transfer of sIPSCs reflects weakened inhibitory control over principal neurons. Remarkably, perineuronal nets, specialized extracellular matrices surrounding PV^+^ interneurons, are also compromised in AD, further destabilizing synaptic function and GABAergic signaling.^152^, ^166^, ^168^

#### 6.2.4 Olfactory dysfunction

Olfactory dysfunction has emerged as a promising early diagnostic indicator for AD, often manifesting during the prodromal stage prior to significant cognitive impairment becoming apparent.^169^ The age‐dependent accumulation of neurotoxic Aβ oligomers follows a well‐defined spatiotemporal trajectory in AD pathogenesis. In murine models, this pathological cascade initiates in the olfactory epithelium at 1–2 months of age, advances to the olfactory bulb (OB) by 3–4 months, and ultimately extends central limbic and cortical regions, including the anterior olfactory nucleus, piriform cortex, and hippocampus by 6–7 months.^170^ Within the OB, GABAergic interneurons show selective vulnerability to Aβ oligomer toxicity. Aβ directly targets GABAergic synapses, triggering axon degeneration and synaptic loss.^130^, ^132^, ^171^ Recent evidence indicates that downregulation of ErbB4 expression in PV^+^ interneurons disrupts GABAergic transmission in AD mice, resulting in hyperexcitability of mitral/tufted cells in the OB.^172^ These circuit‐level impairments accelerate the onset of olfactory deficits in young adult AD models. Notably, quantitative changes in PV^+^ interneuron populations may only manifest during later adulthood in murine models, with PV^+^ cells demonstrating relatively lower vulnerability compared to other neuronal subtypes.^173^, ^174^ Despite its clinical relevance, current research on olfactory dysfunction in AD remains relatively limited, and the underlying pathophysiological mechanisms require further elucidation.

## 7 CONCLUSION

PV^+^ interneurons play a pivotal role in regulating neural network dynamics due to their distinctive cellular architecture and physiological properties. Originating from the MGE, these neurons navigate through the MZ and IZ/SVZ via a precisely orchestrated molecular cascade to reach their final cortical destinations. During the postnatal period, PV^+^ interneurons rapidly integrate into developing neural circuits and undergo protracted maturation processes, including myelination and extensive synaptic pruning, which span several months. Mature PV^+^ neurons exhibit fast‐spiking properties enabled by their specialized dendritic arborization and contribute critically to the generation of gamma oscillations—a metabolically demanding process. A growing body of evidence indicates that impaired PV^+^ interneuron function is a hallmark of AD pathology. The loss of PV^+^ neurons, along with alterations in metabolic protein expression, synaptic dysfunction, E/I imbalance, and downregulation of specific ion channels, is closely associated with AD pathogenesis. Given their essential role in fine‐tuning network activity, PV^+^ interneurons represent a promising therapeutic target for AD.

## AUTHOR CONTRIBUTIONS

**Mengyan Wu**: Writing–original draft, conceptualization, visualization. **Xingdong Zeng**: Writing–original draft, conceptualization. **Yongle Cai**: Writing–original draft. **Haonan Chen**: Writing–original draft. **Qianying Li**: Writing–original draft. **Hao Yang**: Writing–review and editing, supervision, conceptualization.

## CONFLICT OF INTEREST STATEMENT

The authors declare no competing interests.

## ETHICS STATEMENT

Not applicable.

## CONSENT

All authors consent to the submission and publication of this article.

## Data Availability

The data that support the findings of this study are available from the corresponding author upon reasonable request.
